# Effects of Weak Electric Fields on the Denitrification Performance of *Pseudomonas stutzeri*: Insights into Enzymes and Metabolic Pathways

**DOI:** 10.3390/microorganisms12061218

**Published:** 2024-06-17

**Authors:** Xuyan Zhu, Feng Lin, Ji Sun, Xin Li, Guangcan Zhu, Yongze Lu, Liwei Sun, Hongyang Wang

**Affiliations:** 1 School of Energy and Environment, Southeast University, Nanjing 210096, China; xuyanzhu@seu.edu.cn (X.Z.); linf@seu.edu.cn (F.L.); 220210570@seu.edu.cn (J.S.); lix@seu.edu.cn (X.L.); yzlu@seu.edu.cn (Y.L.); liwei-sun@seu.edu.cn (L.S.); 2 Chinese Research Academy of Environmental Sciences, Beijing 100012, China

**Keywords:** weak electric fields, denitrification process, *Pseudomonas stutzeri*, denitrifying enzymes, microbial metabolic pathways

## Abstract

Enhanced denitrification has been reported under weak electric fields. However, it is difficult to investigate the mechanism of enhanced denitrification due to the complex interspecific interactions of mixed-culture systems. In this study, *Pseudomonas stutzeri*, capable of denitrification under anaerobic conditions, was selected for treating low COD/N (2.0, ratio between concentration of chemical oxygen demand and NO_3_^−^-N) artificial wastewater under constant external voltages of 0.2, 0.4, and 0.6 V. The results revealed that *P. stutzeri* exhibited the highest efficiency in nitrate reduction at 0.2 V. Moreover, the maximum nitrate removal rate was 15.96 mg/(L·h) among the closed-circuit groups, 19.39% higher than that under the open-circuit group. Additionally, a notable reduction in nitrite accumulation was observed under weak electric fields. Enzyme activity analysis showed that the nitrate reductase activities were significantly increased among the closed-circuit groups, while nitrite reductase activities were inhibited. Transcriptomic analysis indicated that amino acid metabolism, carbohydrate metabolism, and energy metabolism were increased, enhancing the resistance of *P. stutzeri* to environmental stress and the efficiency of carbon source utilization for denitrification. The current study examined the impacts of weak electric fields on enzyme activities and microbial metabolic pathways and offers valuable insights into the mechanism by which denitrification is enhanced by weak electric fields.

## 1. Introduction

Biological methods applied to treat tailwater from wastewater treatment plants require additional carbon sources [1]. However, this practice compromises water quality and increases operational and maintenance costs [2]. Bioelectrochemical systems (BESs) have been integrated with denitrification, leveraging electrophilic microorganisms to enhance the denitrification process [3,4]. This innovative approach not only improved efficiency but also created new possibilities for energy conservation in wastewater treatment. Therefore, more interest has been generated in exploring the denitrification process under weak electric fields. In a previous study on potentiostatic BESs for treating low COD/N wastewater, although the external voltages were below 1.0 V, the max total nitrogen (TN) removal efficiency in the closed-circuit groups was approximately 3.5 times higher than that in the open-circuit group [5]. Additionally, Qin et al. demonstrated that under a weak external voltage of 0.1 V, the TN removal reached 91.3% [6]. These findings indicate that the denitrification process is markedly enhanced under weak electric fields. Nevertheless, the specific microbiological mechanisms behind this enhancement remain unclear.

There is increasing evidence that the metabolisms of microorganisms are influenced under weak electric fields, consequently impacting system performance. Rodrigo Quejigo et al. illustrated the complete removal of isoproturon in microbial electroremediating cells, where the oxidative metabolism of microorganisms was stimulated by electrodes [7]. Moreover, the production of acetyl-CoA from acetate was facilitated by the elevated intracellular production of NADH in microbes under a low-intensity electric field of 0.05 V [8]. Under low-intensity electric fields, the abundance of genes associated with substrate-binding protein, membrane protein, and phosphate transport system substrate-binding protein was increased, potentially attributed to the enhancement of cell membrane permeability [9]. Thus, exploring the impacts of weak electric fields on microbial metabolisms could elucidate the mechanisms behind the enhanced denitrification.

Changes in intracellular enzyme activities associated with denitrification processes are also significant in the presence of weak electric fields. Parvanova-Mancheva et al. suggested that the influence of an electric field on denitrification might be reflected in alterations in enzyme activity and the regeneration of cofactors [10]. Similarly, She et al. proposed that external microcurrent stimulation could enhance the activity of cell dehydrogenase, thereby augmenting the denitrification rate [11]. Moreover, when investigating coupled sulfur and electrode-driven autotrophic denitrification, an increase in the abundance of genes encoding denitrifying enzymes potentially enhanced denitrification [12]. A weak electric field has been shown to affect nitrate reductase and nitrite reductase activities, resulting in variations in nitrate removal and nitrite accumulation [5]. In addition to weak electric fields, the synthesis of denitrifying enzymes was also affected by the low COD/N, causing unstable enzyme structures and changing activities [13]. Hence, further research is needed to understand the variations in denitrifying enzyme activities under weak electric fields and low COD/N.

Previous studies have explored the mechanisms of enhanced denitrification based on the evolution of microbial communities and interspecific interactions [14,15]. However, in pure culture systems, the mechanisms can be more clearly explored [16,17]. In this study, *P. stutzeri*, prominently featured in a previous biocathode system [18] and capable of complete denitrification under anaerobic conditions [19], was selected. The efficacy of *P. stutzeri* in treating low COD/N artificial wastewater under weak electric fields (constant voltage of 0.2, 0.4, and 0.6 V) was assessed in this study. Moreover, the changes in denitrifying enzyme activities of *P. stutzeri* were examined to elucidate differences in the denitrification process. Furthermore, the results provide insights into the mechanism through which the weak electric fields enhance denitrification in *P. stutzeri* based on transcriptomics analysis.

## 2. Materials and Methods

### 2.1. Experimental Setup

**Figure 1:**
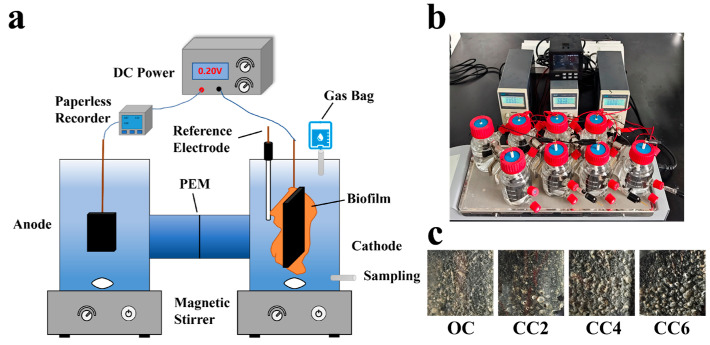
Schematic diagram and operational details of bioelectrochemical systems (BESs), including schematic diagram of BESs (**a**), actual operation diagram of the reactors (**b**), and the biofilms after the first two stages (**c**), detailed in Figure S1.

As depicted in Figure 1a,b, the experimental configuration illustrates a BES reactor comprising two compartments. Each compartment possessed a volume of 250 mL and a headspace of 75 mL. The cathode was constructed using carbon felt measuring 6 cm × 5 cm × 0.5 cm, while the anode was composed of a graphite sheet measuring 3 cm × 2 cm × 0.3 cm. The reference electrodes were Ag/AgCl electrodes. The cathode and anode chambers were separated by a perfluorosulfonic acid-based proton exchange membrane (PEM, Nafion 117, DuPont, Wilmington, DE, USA) with a diameter of 3 cm. The external weak electric fields were controlled by DC power (QJ3003H, Jiuyuan, Ningbo, Zhejiang, China) and divided into an open-circuit group (OC) and 3 closed-circuit groups (CC). The closed-circuit groups were the CC2, CC4, and CC6 groups, operated at constant voltages of 0.2, 0.4, and 0.6 V, respectively. Currents were recorded with a paperless recorder (SIN-R9600, Sinomeasure, Hangzhou, Zhejiang, China).

Catholyte was prepared according to Tables S1 and S2. *P. stutzeri* (CCTCC AB 94052 or ATCC 17588), capable of denitrification under anaerobic conditions [19], was obtained from the China Center for Type Culture Collection (Wuhan, China) for cathode inoculation. The reactors and culture media underwent sterilization, and the catholyte underwent a 30 min aeration with high-purity N_2_ to remove oxygen. High-purity He was utilized for aeration before detecting the composition of the headspace gas. Following the connection of the BESs to a direct current power supply, the reactors were operated at room temperature (25 ± 1 °C), and the magnetic stirrer (MS-M-S10, DLAB, Beijing, China) was adjusted to approximately 100 rpm to ensure proper microbial mass transfer.

### 2.2. Operation of BESs

The operation of the reactors is shown in Table 1. To facilitate the formation of cathodic biofilms, the COD/N of reactors was set to 3.5 during the pre-operational phase (Stages I and II), with NaAc and NO_3_^−^-N concentrations of 292 and 65 mg/L, respectively. Furthermore, a batch cycle time of 5 days and 3 days was implemented during Stages I and II, respectively. After establishing the stable biofilms on the surface of the carbon felts (Figure 1c), the solution in all reactors was completely replaced for subsequent experiments (Stage III) using a freshly prepared anaerobic medium. The COD/N of the catholyte was adjusted to 2.0, with NaAc and NO_3_^−^-N concentrations of 167 and 65 mg/L, respectively.

### 2.3. Analytical Methods

#### 2.3.1. Chemical Measurements

The determinations of NO_3_^−^-N, NO_2_^−^-N, and NH_4_^+^-N in water samples were performed using the automatic discrete analyzer (Cleverchem Anna, DeChem-Tech, Hamburg, Germany). According to a previous study, the linear fittings of the maximum nitrate removal rate in all groups were analyzed using the software Origin (version 2022b, OriginLab Corporation, USA), where the slope represents the nitrate removal rate and R^2^ represents the degree of fit [20]. The closer the R^2^ is to 1, the better the fitted curve represents the real situation. pH changes in the cathode solution were monitored using a pH meter (PH100, YSI, Yellow Springs, OH, USA).

The composition of the headspace gas in the reactor was determined using a gas chromatograph (GC3900, Rui-neng, Dongying, Shandong, China) equipped with a thermal conductivity detector (TCD). A 500 μL gas sample was extracted and separated using a molecular sieve 5A column and a TDX-01 column. The temperatures of the injection port, column oven, and TCD were set to 80 °C, 100 °C, and 120 °C, respectively. The column flow rate was 0.9 mL/min, with a split ratio of 30:1. The analyses were performed under conditions of an air flow rate of 400 mL/min and a hydrogen flow rate of 40 mL/min. The contents of NaAc in the cathode chamber were measured using a gas chromatograph (GC7890B, Agilent, Santa Clara, CA, USA) equipped with flame ionization detection.

The fraction (*η*) of electrons supplied by the carbon source that were used for nitrate reduction was obtained using Equation (1).

(1) $$\eta \;(\%)=\frac{E_{N_{2}}{+E}_{{NO}_{2}^{-}\;}{+E}_{{NH}_{4}^{+}}}{E_{NaAc}}\;\times \;100\%$$

where *E_NaAc_* is the number of electrons supplied by the utilized NaAc, mmol; $E_{N_{2}}$ is the number of electrons required to reduce nitrate to N_2_, mmol; $E_{{NO}_{2}^{-}\;}$ is the number of electrons required to reduce nitrate to nitrite, mmol; and $E_{{NH}_{4}^{+}}$ is the number of electrons required to reduce nitrate to NH_4_^+^-N, mmol.

#### 2.3.2. Determination of Denitrification Reductase Activity

The impact of weak electric fields on microbial activity was assessed by detecting the nitrate reductase and nitrite reductase (NR and NiR, respectively) of *P. stutzeri*. Biofilms from the cathodes (11.0 cm^2^ in every reactor) were collected at 2 h after the reactor was initiated and washed three times in a 0.01 M phosphate buffer solution (PBS). Afterward, the biofilms were resuspended in a 0.01 M PBS solution and then crushed for 3 min using an ultrasonic crusher (XO-650D, Atpio, Nanjing, China) with an ice bath. After being centrifugated in a freezing centrifuge (10,000× *g*, 4 °C) for 10 min, the supernatant (crude enzyme solution) was collected and stored on ice. NR and NiR activities of the crude enzyme solution were determined using specific assay kits (Beijing Boxbio Science & Technology Co., Ltd., Beijing, China) following established protocols. The calculations were performed as displayed in Formulas (2) and (3). Protein concentrations of the crude enzyme solution were determined using the Bradford method with modifications for enhanced accuracy [21].

(2) $$\mathrm{NR}\;\left(U/\mathrm{mg}\;\mathrm{prot}\right)=\frac{\Delta {A\;\times \;V}_{act}{\;\times \;10}^{6}}{{\varepsilon \;\times \;d}_{1}{\;\times \;V}_{samp}{\;\times \;Cpr\;\times \;T}_{1}}$$

(3) $$\mathrm{NiR}\;(U/\mathrm{mg}\;\mathrm{prot})=\frac{{x\;\times \;V}_{\mathrm{ez}}}{V_{\mathrm{samp}}{\;\times \;\mathrm{Cpr}\;\times \;T}_{2}}$$

Notes: ∆*A*: the difference in absorbance before and after the reaction; *V_act_*: the total volume of the reaction system, 2 × 10^−4^ L; *V_samp_*: the volume of the crude enzyme solution added to the reaction system, 0.02 mL; *V_ez_*: the total volume of the enzymatic reaction, 0.14 mL; *x*: the concentration of NO_2_^−^-N measured in μmol/mL; *T*_1_ and *T*_2_: the reaction time, 0.5 h and 1 h, respectively; *ε*: the NADH molar extinction coefficient, 6220 L/mol/cm; *d*_1_: the optical diameter of the 96-well UV plate, 0.5 cm; *Cpr*: the concentrations of protein in the sample, mg/mL; U (NR): the amount of NADH consumption per hour per mg protein; U (NiR): the amount of NO_2_^−^-N reduction per hour per mg protein.

#### 2.3.3. RNA-Seq Analysis

RNA-Seq technology was utilized in this investigation to unveil the transcriptional profile of *P. stutzeri* with and without electrical stimulation. Biofilms of all reactors were collected at 2 h after the reactor was initiated using a sterile scraper in a super-clean workbench, aiming to elucidate the influence of weak electric fields on the metabolic pathways of *P. stutzeri*. Subsequently, these biofilm samples were moved to a sterile tube, promptly placed in liquid nitrogen for rapid freezing, and then transported to Majorbio Biotechnology Co., Ltd. (Shanghai, China) for further analysis.

A RNeasy Mini kit (Qiagen, Düsseldorf, Germany) was used to extract total RNA, with quality assessment performed using an Agilent bioanalyzer. rRNA was removed to enrich mRNA, and fragmentation was achieved with a dedicated buffer. Random hexamers and cleaved mRNA were employed for the initiation of first-strand cDNA synthesis. The library was sequenced on an Illumina Novaseq 6000 instrument (Illumina, San Diego, CA, USA). The resultant library has been cataloged in the Sequence Read Archive with accession number PRJNA1048846. Transcriptome sequencing data were analyzed on the Majorbio Cloud Platform online (www.majorbio.com, accessed on 12 September 2023), encompassing cleansing procedures with SeqPrep (Version 2011) and Sickle (Version 1.33) software, de novo assembly facilitated by Trinity (Version 2.15.0) software, and annotation sourced from various databases (NR, Version 2022.10; Swiss-Prot, Version 2022.10; Pfam, Version 35.0; COG, www.ncbi.nlm.nih.gov/COG/, accessed on 10 August 2023; GO, Version 2022.0915; and KEGG, Version 2022.10). Quantification of transcripts, identification of differentially expressed genes (DEGs), and analyses of functional and pathway enrichments employed RSEM (Version 1.3.3), DESeq2 (Version 1.24.0), and Goa tools (Version 0.6.5) software, respectively, with a fold change (FC) threshold of ≥2 and a significance level of *p* < 0.05 used as criteria for identifying DEGs.

### 2.4. Statistical Analysis

All the analyses were conducted with three replicates to ensure statistical robustness. The means and standard deviations of all the results were calculated using Microsoft Excel 2021 software (Microsoft Corporation, USA). Student’s *t* test was used to assess the significance of changes in results, and *p* value < 0.05 was chosen as the significant value.

## 3. Results and Discussions

### 3.1. Effect of Weak Electric Fields on Denitrification of P. stutzeri

After the first two stages (Stage I and II), the biofilms adhered to the cathode in each reactor matured (Figure S1), then the COD/N of the cathode solution was adjusted to 2.0 in Stage III for further experiments (Figure S2).

**Figure 2:**
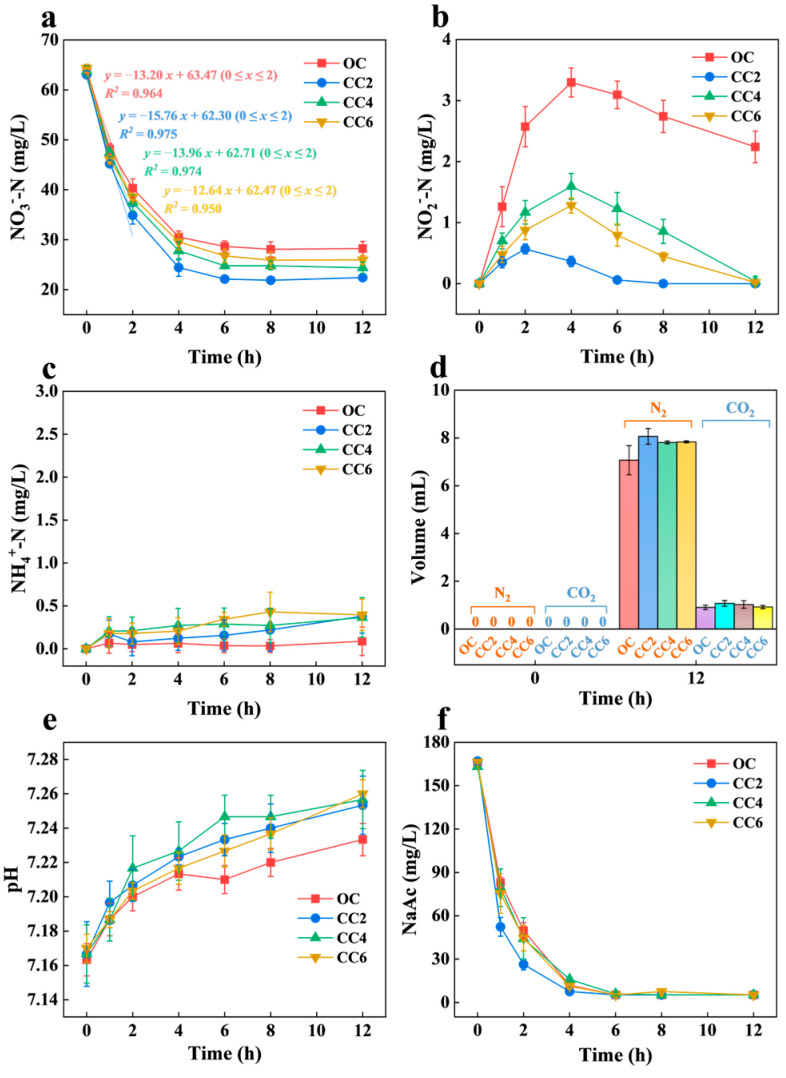
Impacts of weak electric fields on denitrification performance of *P. stutzeri*, including variations in nitrate (NO_3_^—^-N) (**a**), nitrite (NO_2_^—^-N) (**b**), ammonia (NH_4_^+^-N) (**c**), headspace gas composition in the cathode chamber (**d**), pH changes (**e**), and NaAc concentrations (**f**).

Comparative analyses of denitrification performance across various reactors indicated that the CC2 group exhibited the most efficient treatment. As shown in Figure 2a, the concentrations of NO_3_^−^-N in the OC, CC2, CC4, and CC6 groups were reduced to 28.23 ± 1.42, 22.41 ± 0.37, 24.37 ± 1.11, and 25.97 ± 0.87 mg/L, respectively. Compared with the OC group, the reduction efficiencies of NO_3_^−^-N in the closed-circuit groups (CC2, CC4, and CC6) were increased by 15.10%, 10.31%, and 6.45%, respectively. Moreover, the nitrate removal rates in the closed-circuit groups consistently outperformed those in the OC group. During the first 2 h, the CC2 and CC4 groups exhibited an increase of 19.39% and 5.76%, respectively, compared to the nitrate removal rate observed in the OC group (13.20 mg/(L·h)). Wang et al. reported a 13.66% increase in the maximum nitrate removal rate in the closed-circuit group compared to the open-circuit group [22], which was the same as this study. These results suggest that NO_3_^−^-N removal was enhanced under a weak electric field, which is consistent with the results of previous studies [23,24].

Under the condition of a limited electron donor (low COD/N), reduced electron supply and weak electron competition of nitrite reductase could constrain the rate of nitrite reduction, leading to nitrite accumulation [25]. Varied peak NO_2_^−^-N accumulation across each group is demonstrated in Figure 2b. At hour 2, the CC2 group reached a peak value of 0.57 ± 0.08 mg/L, while the OC, CC4, and CC6 groups reached peak values of 3.30 ± 0.24, 1.59 ± 0.21, and 1.28 ± 0.12 mg/L, respectively, at hour 4. At the end of the experiment, the concentration of NO_2_^−^-N in the OC group stabilized at approximately 2.24 ± 0.26 mg/L, whereas the closed-circuit groups exhibited no NO_2_^−^-N. This result indicates that the accumulation of nitrite decreased with increasing voltage. Yang et al. also found that when using a bioelectrochemical system to treat nitrate wastewater, an appropriate electric current (I ≤ 30 mA) was beneficial for nitrate removal. However, too high an electric current (I = 40 mA) would inhibit nitrate removal and result in significant accumulation of NO_2_^–^-N and NH_4_^+^-N [26]. In addition, minor NH_4_^+^-N accumulation (peak < 0.45 mg/L) was observed in the closed-circuit groups, while no accumulation was observed in the OC group (Figure 2c). This suggests that microbial death may be induced by weak electric fields, leading to the release of intracellular NH_4_^+^-N [27], or that NH_4_^+^-N synthesis was activated through the assimilatory pathway [28].

The headspace gas composition of each reactor (Figure 2d) revealed the presence of N_2_ and CO_2_, indicating the denitrification process. Denitrification utilizing organic matter is an alkalinity-producing process, generating 3.57 mg of alkalinity for every 1 mg of nitrate reduction. The voltage applied in this study failed to elevate alkalinity through electrolyzing water for hydrogen production [29]. Ren et al. found that during the heterotrophic anode denitrification process, the pH of the anodic electrolyte consistently increased due to the consumption of protons (H^+^) during nitrate reduction [30]. Consequently, the higher alkalinity in the closed-circuit groups compared to the OC group indicated that an enhanced denitrification process was found in the closed-circuit groups (Figure 2e).

*P. stutzeri* relies on organic carbon sources as electron donors during denitrification. As illustrated in Figure 2f, the carbon source (NaAc) utilization of *P. stutzeri* differed under varying electric fields. The closed-circuit groups exhibited relatively higher rates of carbon source consumption compared to the OC group, with the CC2 group consuming over 110 mg/L of the carbon source in the initial hour, which was higher than that observed in the other groups. These results imply that the carbon source utilization rate of *P. stutzeri* was enhanced under 0.2 V, resulting in the acceleration of the denitrification process. Zhu et al. also found that electricity promoted organic decomposition, increasing COD removal and generating more electrons for cathodic denitrification reactions [31], which is consistent with the results of this study.

**Figure 3:**
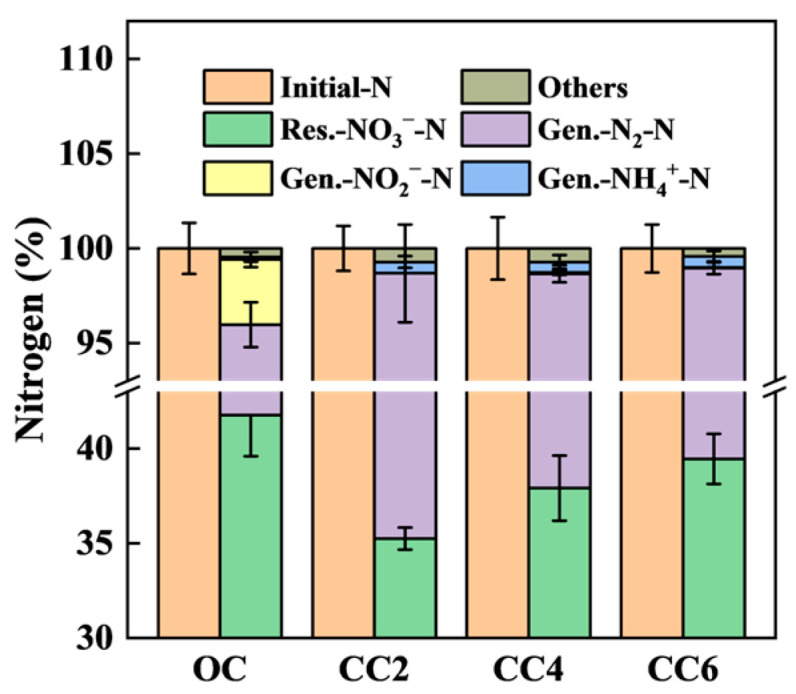
Nitrogen balance calculation with and without weak electrical stimulations. ‘Res.’ and ‘Gen.’ represent the ‘residual’ and ‘generated’ nitrogen, respectively. The N_2_O production was included in ‘Others’.

The calculation of the nitrogen balance (Figure 3) revealed that the initial nitrate was primarily converted into N_2_ via biological denitrification, with nitrite accumulation observed in the OC group. However, minor residual NH_4_^+^-N was present in the closed-circuit groups. These results are consistent with previous findings.

### 3.2. Denitrification Enzyme Activities and Electron Utilization of P. stutzeri

In contrast to the OC group, the performance of *P. stutzeri* was enhanced under weak electric fields during the initial two denitrification phases, namely nitrate and nitrite reduction. To explore the mechanism, the activities of nitrate reductase and nitrite reductase (NR and NiR, respectively), along with electron utilization by *P. stutzeri*, were studied.

**Figure 4:**
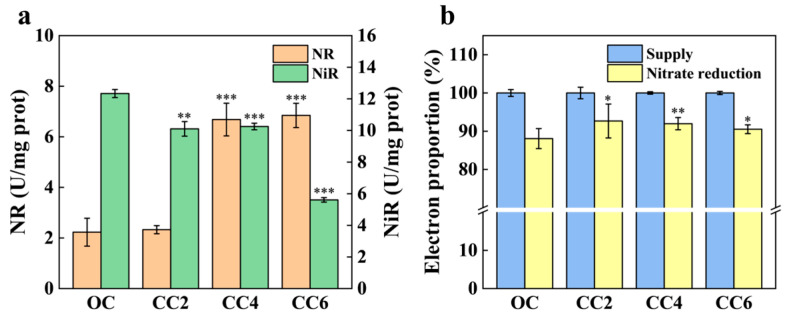
Variations in nitrate reductase and nitrite reductase activities (**a**) and electron utilized for nitrate reduction (**b**) with and without weak electrical stimulation. *, ** and *** represent *p* value < 0.05, 0.01, and 0.001 when compared with OC group, respectively.

The activities of NR and NiR are critical in denitrification; they determine the efficiency and rate of denitrification, consequently influencing nitrate and nitrite concentrations in the effluent [32]. As illustrated in Figure 4a, the NR and NiR activities of *P. stutzeri* were differentially affected by the weak electric fields. The NR activities for CC2, CC4, and CC6 groups were 4.48%, 199.55%, and 206.73% higher than those observed in the OC group (2.23 ± 0.55 U/mg prot), respectively, while the NiR activities were suppressed under weak electric fields, showing reductions of 22.18%, 16.94%, and 54.54%, respectively, compared to the OC group (12.34 ± 0.25 U/mg prot). Despite lower NiR activities in the closed-circuit groups compared to the OC group, little nitrite was accumulated (Figure 2b). As illustrated in Table S3, the total protein contents of the CC2, CC4, and CC6 groups increased by 11.12%, 10.04%, and 115.02%, respectively, compared to the OC group, indicating no apparent impact of the weak electric fields on the nitrite reduction process. As shown in Figure 4b, 93% of electrons acquired from the carbon source in the CC2 group were allocated for nitrate reduction, indicating that the electron utilization of the CC2 group was highly efficient.

### 3.3. Transcriptomic Analysis of P. stutzeri

According to the transcriptomic data, a substantial dataset of raw reads, ranging from 25,012,644 to 32,716,588, was initially acquired from all samples. After filtering and trimming procedures, the clean reads exhibited high-quality metrics, with Q20 and Q30 values ranging from 98.57% to 98.70% and from 95.54% to 95.95%, respectively. These results affirmed the robust quality of the transcriptomic sequencing data for *P. stutzeri*, ensuring their reliability for subsequent in-depth analysis.

#### 3.3.1. Annotation and Enrichment of Differentially Expressed Genes

**Figure 5:**
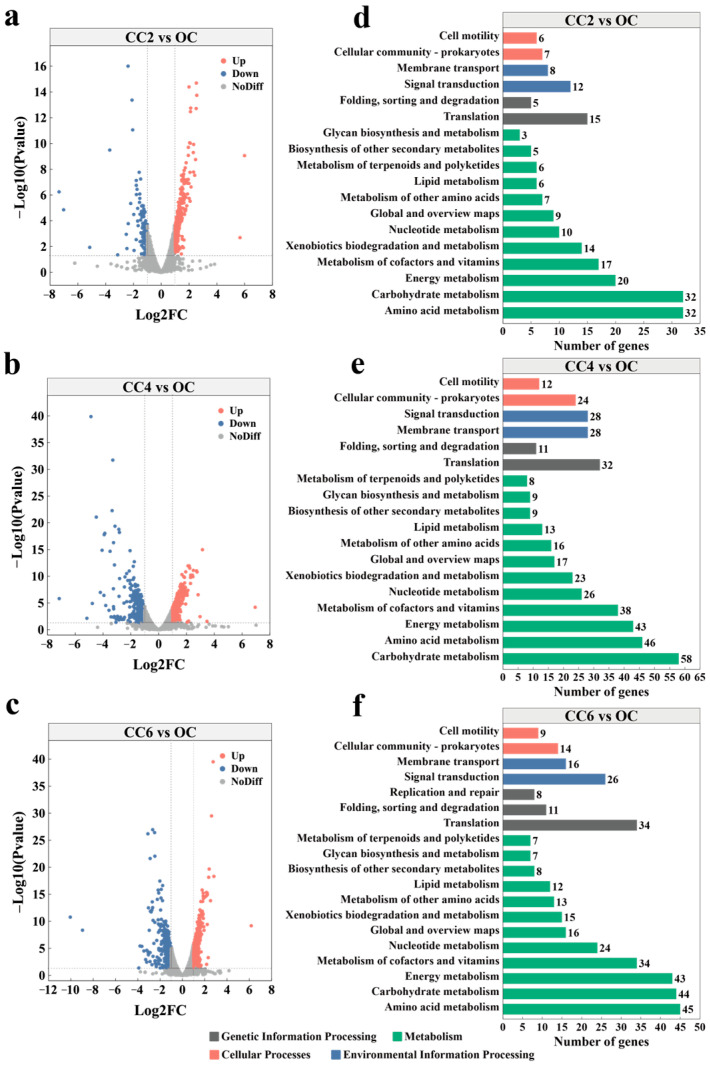
Volcano plots illustrating (**a**–**c**) differentially expressed genes (DEGs) in the closed-circuit groups compared with the OC group. Panels (**d**–**f**) display the annotation of KEGG pathways for DEGs in the CC2, CC4, and CC6 groups, respectively, compared to the OC group.

The transcriptomic findings suggested an impact of weak electric fields on the transcriptional profile of *P. stutzeri*, influencing key metabolic pathways and cellular functions. In this paper, the OC group served as a control, and the closed-circuit groups were divided into three subgroups, namely CC2 vs. OC, CC4 vs. OC, and CC6 vs. OC. Comparative analyses revealed distinct patterns in gene expression under weak electric fields. At 0.2 V (Figure 5a), 308 genes were up-regulated, while 173 genes were down-regulated. The CC4 group exhibited 798 differentially expressed genes (DEGs) (Figure 5b), of which 401 were up-regulated and 397 were down-regulated. Similarly, the CC6 group displayed 745 DEGs (Figure 5c), comprising 365 up-regulated and 380 down-regulated genes.

To gain insights into the functional implications of these DEGs, two major databases (COG and KEGG) were selected for functional enrichment analysis. The COG database, focusing on phylogenetic classifications of proteins across genomes [33], revealed 1639 genes mapped to 23 functional classes. The functional categories included translation, amino acid transport, metabolism, energy production, and conversion, among others (Figure S3).

In KEGG pathway analysis, the CC2, CC4, and CC6 groups were annotated with varying numbers of genes to 34, 38, and 37 pathways, respectively. The metabolism category encompassed 54.15% to 59.54% of genes in each group, with amino acid metabolism, carbohydrate metabolism, and energy metabolism dominating (Figure 5d–f). As shown in Figure S4, the KEGG metabolic pathways in the closed-circuit groups, compared to the OC group, were concentrated on ribosome, styrene degradation, glyoxylate and dicarboxylate metabolism, tyrosine metabolism, phenylalanine metabolism, and pyruvate metabolism. These findings reveal the potential adaptations of *P. stutzeri* to weak electric fields.

#### 3.3.2. Metabolic Mechanism of *P. stutzeri* under Weak Electric Fields

**Figure 6:**
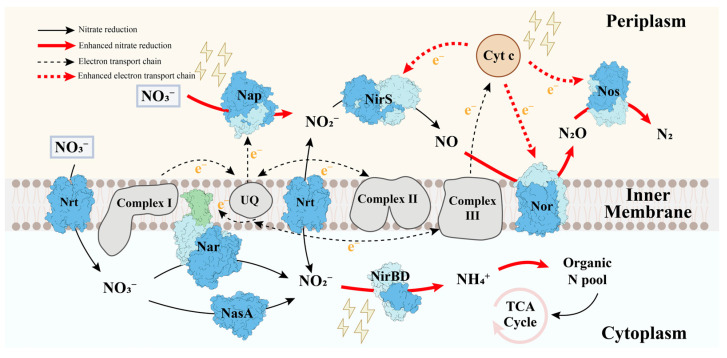
The impacts of weak electric fields on the process of denitrification in *P. stutzeri*.

The denitrification process involves a series of biological reactions that necessitate energy and electron consumption [34]. The impact of weak electric fields on denitrification was examined to elucidate the denitrification mechanism of *P. stutzeri* (Figure 6).

The impact of weak electric fields on denitrification is illustrated in Figure 6. The enhanced nitrate reduction processes were mainly attributed to the significant increase in NR activities in the CC4 and CC6 groups (Figure 4a). However, NR activity was only slightly improved (4.48% higher than the OC group) in the CC2 group. According to the transcriptomic results, the nitrate reduction in the CC2 group was attributed to the nitrate ABC transporter permease (*nrtB*) and assimilatory nitrate reductase (*nasA*). As illustrated in Figure 6 and Figure S5, nitrate was transported to the cytoplasm due to the up-regulation of *nrtB* and was then reduced by the up-regulated *nasA* [35], which was the main nitrate reduction pathway in the CC2 group.

Enhanced enzyme activity is typically attributed to the increased abundance of corresponding genes [36,37]. As discussed earlier, notably enhanced NR activities were observed in the CC4 and CC6 groups. Transcriptomic analysis (Figure S5) showed that the genes encoding periplasmic nitrate reductase (*napAB*) and membrane-bound nitrate reductase (*narGHI*) were detected in all groups. Given the significantly higher abundance of *napAB* than that of *narGHI* (Figure S5), periplasmic nitrate reductase could play a critical role. However, no significant difference in the abundance of *napAB* in *P. stutzeri* under the closed-circuit groups compared to the OC group was observed. According to previous research, two hypotheses for enhancing NR activity have been proposed. On the one hand, the redox potential of [4Fe-4S] clusters, responsible for the electron transfer chain, might be altered under weak electric fields, affecting intracellular electron transfer kinetics and influencing enzyme activity [38]. On the other hand, considering the role of metal Mo, located at the active center, in cleaving the N-O bond of nitrate, the structure of the active site in nitrate reductase could also be changed, affecting enzyme activity [39]. Further research is warranted to explain this phenomenon, where denitrifying enzyme activity was noticeably boosted under weak electric fields without notable increases in the abundance of corresponding genes.

Generally, the abundance of enzymes was controlled by the abundance of corresponding genes [40]. The abundance of genes encoding NiR was significantly down-regulated under weak electric fields, explaining the suppressed NiR activity (Figure 4a). However, higher total protein contents in the closed-circuit groups indicated no apparent impact of the weak electric fields on the nitrite reduction process (Figure 4b). Additionally, the abundance of genes encoding nitrite reductase (*nirBD*), involved in controlling the nitrite assimilatory pathway [28], was up-regulated in the closed-circuit groups (Figure S5). This suggests that partial nitrite was reduced to ammonia via assimilation, then entering the ‘Organic N pool’ [41], resulting in minor ammonia accumulation in the closed-circuit groups (Figure 2c). Additionally, the up-regulation of cytochrome c (Figure S5) indicated that more electrons were transferred to the reduction of NO and N_2_O, playing a significant role in the synergistic effects [42].

**Figure 7:**
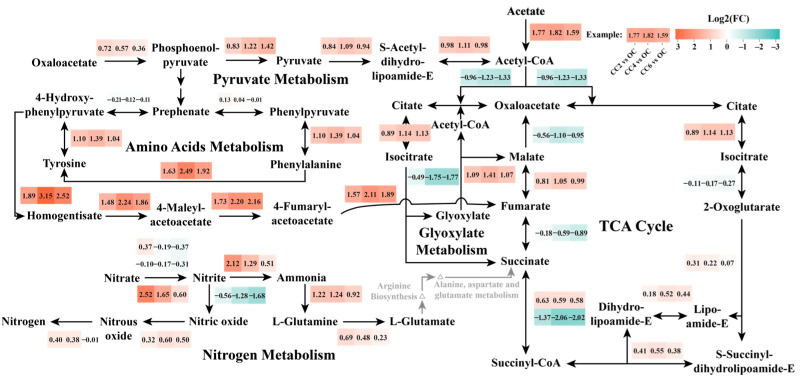
Mechanism of external weak electric fields influencing metabolic pathways of *P. stutzeri*, depicting the intricate mechanism by which external weak electric fields influence various metabolic pathways in *P. stutzeri*. The red between pathways indicates the up-regulation of gene expression levels in the closed-circuit groups (CC2, CC4, and CC6) compared with the OC group, while green indicates down-regulation. Specific gene information for each pathway is provided in Supplementary Material Figure S5.

Annotated information on the DEGs indicates that amino acid metabolism and carbohydrate metabolism pathways were primarily stimulated in *P. stutzeri* under weak electric fields (Figure 7).

Amino acid metabolism regulates substance transport and the synthesis of key cellular enzymes and affects pollutant degradation within cells [43]. Figure 7 demonstrates the connection between amino acid metabolism, a critical component of protein function, and the denitrification process through the TCA cycle [44]. When exposed to the external environment, *P. stutzeri* relies on tyrosine degradation to maintain its energy supply [45]. Notably, the abundance of genes related to metabolism from tyrosine to fumarate was up-regulated in the closed-circuit groups. Subsequently, the final product, fumarate, underwent the TCA cycle and produced NADH, which served as an electron donor for denitrification [46]. Consequently, the proportions of the electron used for nitrate reduction in the closed-circuit groups were higher than those found in the OC group. Amino acids also contribute to stabilizing intracellular membranes and proteins, aiding *P. stutzeri* in resisting environmental stressors and ensuring normal microbial metabolism [47].

Carbohydrate metabolism is crucial for cell energy production and storage [48]. In the closed-circuit groups, *P. stutzeri* was induced to enhance the abundance of genes involved in the TCA cycle, glyoxylate metabolism, and pyruvate metabolism, including aconitate hydratase (EC: 4.2.1.3; PSTAB_1744), 2-oxoglutarate dehydrogenase (EC: 1.2.4.2; PSTAB_1772), lipoamide dehydrogenase (EC: 1.8.1.4; PSTAB_1774), dihydrolipoyl dehydrogenase (EC: 2.3.1.61; PSTAB_1773), succinyl-CoA synthetase (EC: 6.2.1.5; PSTAB_1776), fumarate hydratase (EC: 4.2.1.2; PSTAB_1591), malate synthase (EC: 2.3.3.9; PSTAB_3804), aconitate hydratase (EC: 4.2.1.3; PSTAB_1744), pyruvate dehydrogenase (EC: 2.3.1.12; PSTAB_2903), pyruvate kinase (EC: 2.7.1.40; PSTAB_1054), and phosphoenolpyruvate carboxylase (EC: 4.1.1.31; PSTAB_2731). Sodium acetate was converted to acetyl-CoA to enter the TCA cycle, ultimately providing energy for denitrification. According to the transcriptomic results, the abundance of genes encoding acetyl-CoA synthetase in the closed-circuit groups was 3.02~3.53 times higher than that observed in the OC group, consequently enhancing the utilization of substrate (Figure 4b). NADH produced during the TCA cycle entered the electron transfer chains, underwent oxidative phosphorylation, and generated substantial ATP. The up-regulation of ATP-producing enzymes, such as pyruvate kinase, indicated that *P. stutzeri* was stimulated to generate more energy under weak electric fields, facilitating denitrification [49]. Additionally, glyoxylate and dicarboxylate metabolism, as well as pyruvate metabolism, were significant metabolic pathways in carbohydrate metabolism in *P. stutzeri*, which shared similar functions in sustaining carbon homeostasis and creating energy [43].

In conclusion, notable up-regulation of gene expression encoding enzymes involved in both amino acid metabolism and carbohydrate metabolism was observed in *P. stutzeri* under the closed-circuit groups. The results indicate the capacity of *P. stutzeri* to withstand environmental stress and sustain normal metabolic pathways in the face of external fluctuations. Moreover, the findings imply that the application of weak electric fields could accelerate energy generation, such as ATP and NADH, consequently expediting the denitrification process.

## 4. Conclusions

Denitrification, primarily the reduction of nitrate, was enhanced in the closed-circuit groups. Transcriptomic analysis indicated the up-regulation of genes involved in amino acid metabolism, pyruvate metabolism, and the TCA cycle, revealing increased carbon source utilization for denitrification under weak electric fields. Notably elevated NR activities were observed in the CC4 and CC6 groups without the corresponding up-regulation of gene abundance. In the future, further investigation of this intriguing result could be conducted with molecular dynamic simulation software such as AMBER or GROMACS.

## Figures and Tables

**Table 1:** The operation of the reactors.

Stage	COD/N	Batch Cycle Time (Days)
I	3.5	5
II	3.5	3
III	2.0	3

## Data Availability

Data are contained within the article and Supplementary Materials.

## Supplementary Materials

The following supporting information can be downloaded at: https://www.mdpi.com/article/10.3390/microorganisms12061218/s1, Figure S1: Growth changes of cathodic biofilms in each group throughout the entire operation; Figure S2: Denitrification performance of reactors (A, B, C, and D represent OC, CC2, CC4, and CC6, respectively) throughout the entire operation, where RE represents Removal Efficiency; Figure S3: COG function information annotated by DEGs in closed-circuit groups (CC2, CC4, and CC6) compared with the open-circuit group; Figure S4: Enrichment of KEGG pathways in DEGs of the closed-circuit groups (CC2, CC4, and CC6) compared with the open-circuit group; Figure S5: The specific information of genes in each pathway (Figure 7) of the closed-circuit groups (CC2, CC4, and CC6) compared with the open-circuit group. *, ** and *** represent *p* value < 0.05, 0.01, and 0.001 when compared with OC group, respectively; Table S1: Components of anolyte and catholyte; Table S2: Components of trace element solution; Table S3: Calculation of total protein content in each reactor.
