# Supplementary material for: Effects of Weak Electric Fields on the Denitrification Performance of Pseudomonas stutzeri: Insights into Enzymes and Metabolic Pathways
Source: Microorganisms. 2024 Jun 17;12(6):1218. doi: 10.3390/microorganisms12061218 (PMC11205929; doi:10.3390/microorganisms12061218)
Supplement: Supplementary file 1 [file microorganisms-12-01218-s001.zip › microorganisms-2970890-supplementary.pdf]

Supplemental Information

Effects of Weak Electric Fields on the Denitrification Performance of *Pseudomonas stutzeri*: Insights into Enzymes and Metabolic Pathways

Xuyan Zhu[a], Feng Lin[a], Ji Sun[a], Xin Li[a], Guangcan Zhu[a] *, Yongze Lu[a], Liwei Sun[a] and Hongyang Wang[b] *

[a] School of Energy and Environment, Southeast University, Nanjing 210096, China

[b] Chinese research academy of environmental sciences, Beijing 100012, China

* Corresponding authors: gc-zhu@seu.edu.cn (G. Z.), wanghongyang_why@126.com (H. W.)

Table S1 Components of anolyte and catholyte

	Description	Content (g/L)
Anolyte	KH_2PO_4	0.816
	K_2HPO_4	5.92
Catholyte	KH_2PO_4	0.816
	K_2HPO_4	5.92
	$NaNO_3$	0.40
	NaAc	0.30/0.17
	$MgCl_2$	0.20
	$CaCl_2$	0.05
	Trace element solution	1.00 mL/L

Table S2 Components of trace element solution

Description	Content (g/L)
$FeCl_3 \cdot 6H_2O$	1.50
$MnCl_2 \cdot 4H_2O$	0.12
$CoCl_2 \cdot 6H_2O$	0.15
$ZnSO_4 \cdot 7H_2O$	0.12
$CuSO_4 \cdot 5H_2O$	0.03
$Na_2MoO_4 \cdot 2H_2O$	0.06
KI	0.18
EDTA	10.00
H_3PO_3	0.15

Table S3 Calculation of total protein content in each reactor

	OC	CC2	CC4	CC6
Total biofilm weight (mg)	424	470	513	422
Protein content (mg/L) [1]	61.32	68.14	67.01	131.85
Total protein content (mg/L)	395.79	439.81	435.52	851.03

[1] Protein content was determined when measuring enzyme activity.

The calculation is below (take OC as an example):

$$\text{Total protein content}=\frac{424}{\frac{424}{60}\times 11}\times 61.32+61.32=\frac{60}{11}\times 61.32+61.32=395.79\ \text{mg/L}$$

Where the total biofilm area is 60 cm^2, while the area of the biofilm measured for enzyme activity is 11 cm^2.

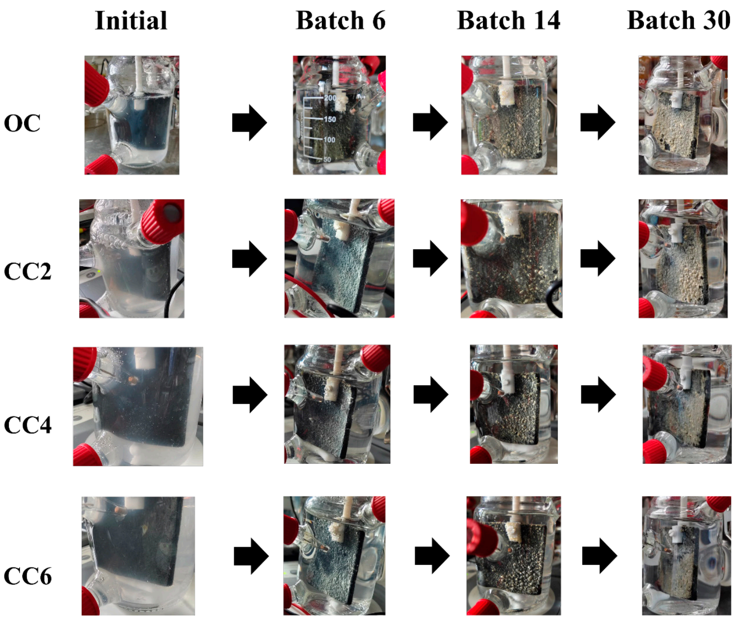

Figure S1 Growth changes of cathodic biofilms in each group throughout the entire operation

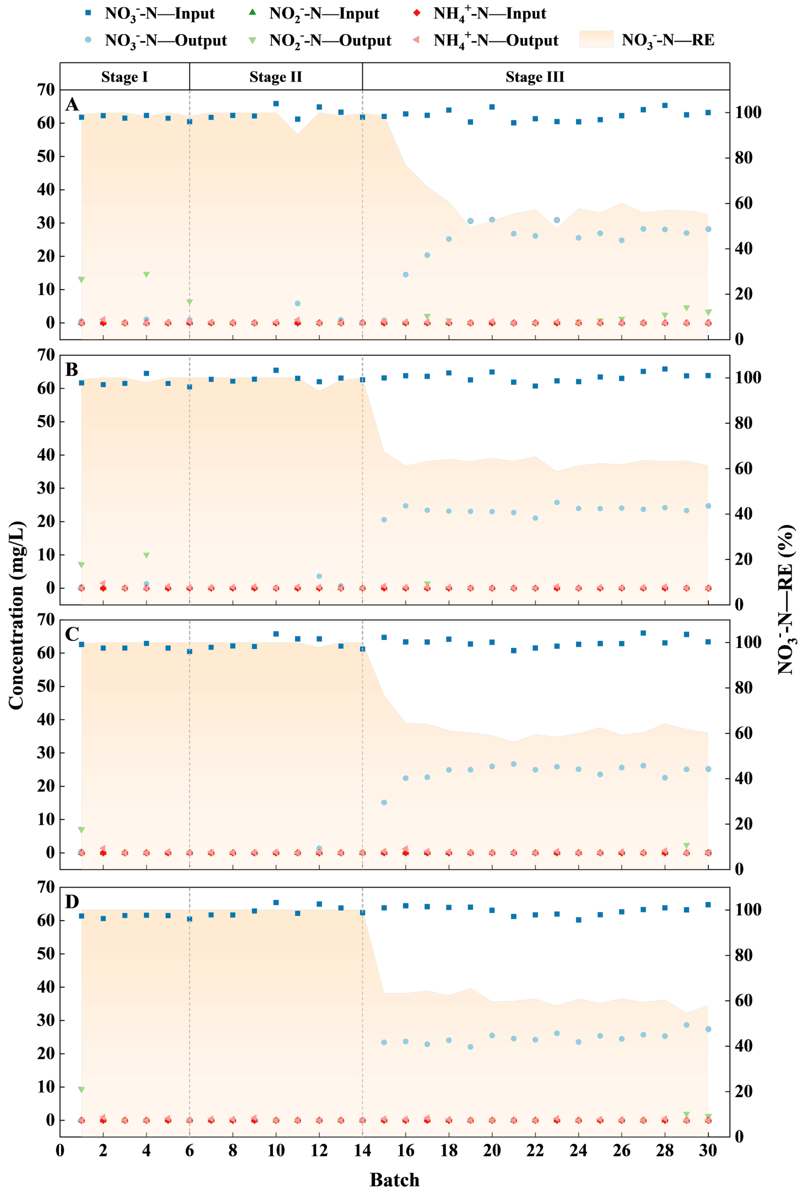

Figure S2 Denitrification performance of reactors (A, B, C, and D represent OC, CC2, CC4, and CC6, respectively) throughout the entire operation, where RE represents Removal Efficiency.

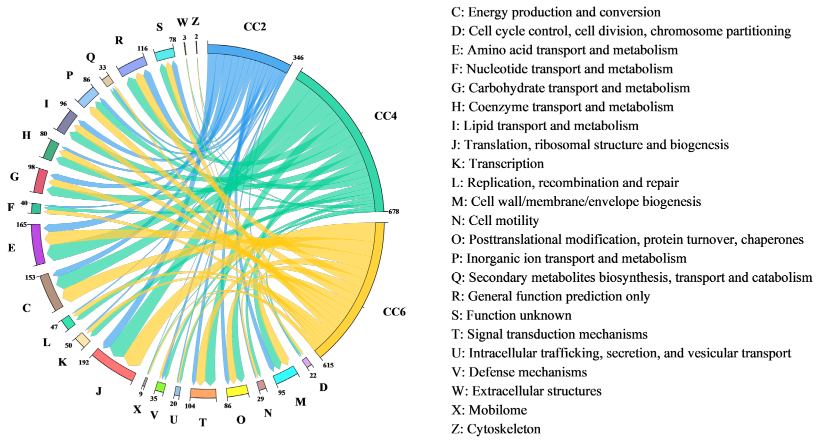

Figure S3 COG function information annotated by DEGs in closed-circuit groups (CC2, CC4, and CC6) compared with the open-circuit group.

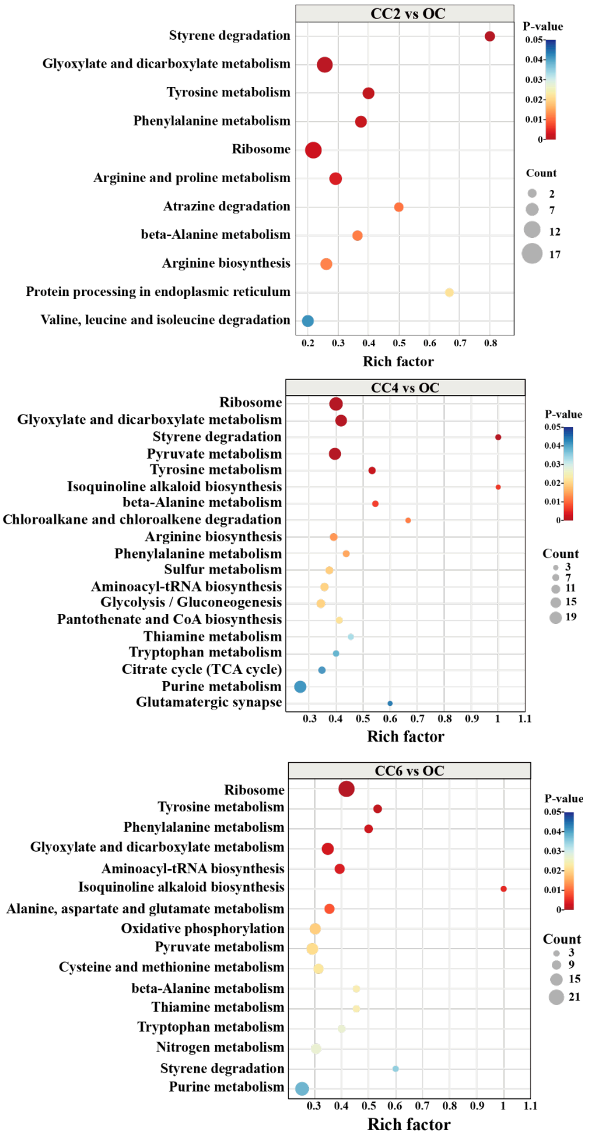

Figure S4 Enrichment of KEGG pathways in DEGs of the closed-circuit groups (CC2, CC4, and CC6) compared with the open-circuit group.

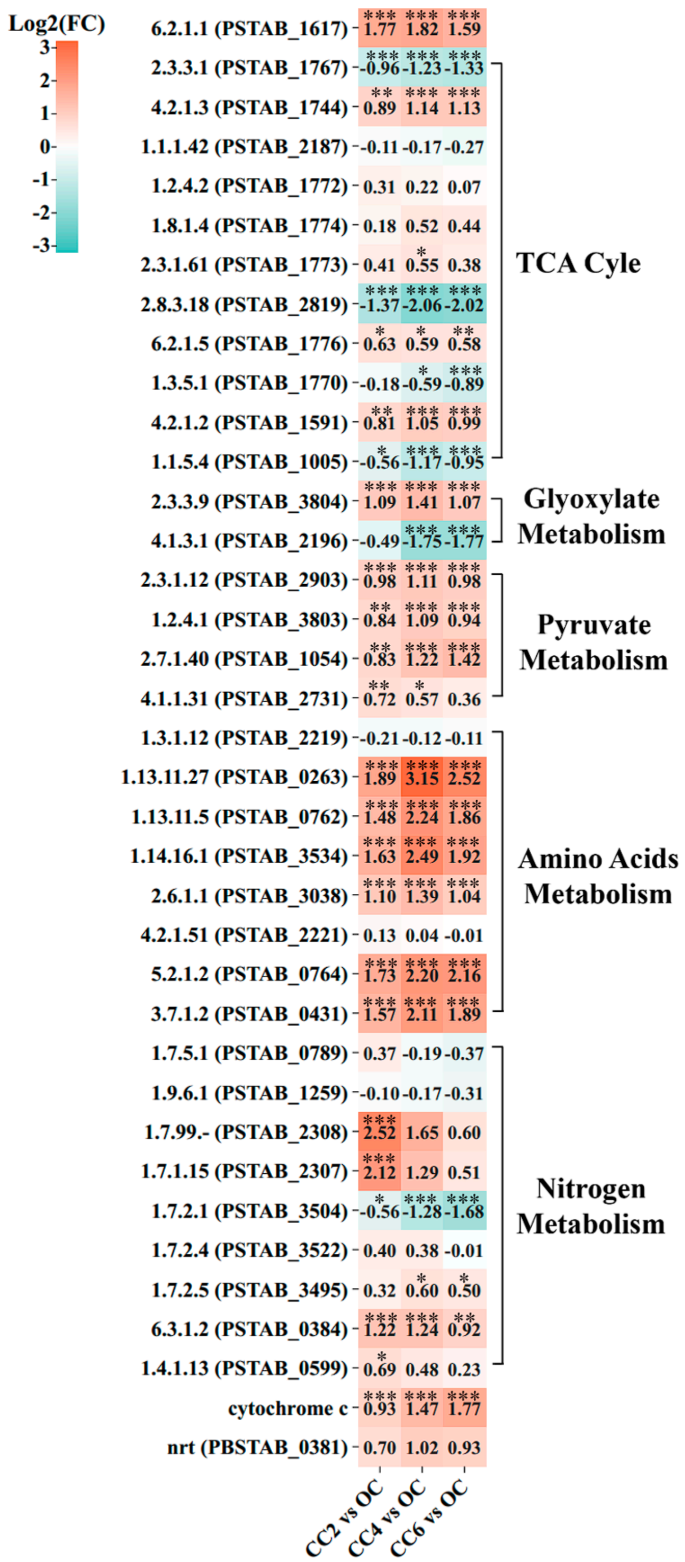

Figure S5 The specific information of genes in each pathway (Fig. 7) of the closed-circuit groups (CC2, CC4, and CC6) compared with the open-circuit group. *, ** and *** represent *P* value < 0.05, 0.01 and 0.001 when compared with OC group, respectively.
